# Plasminogen Activator Inhibitor-1 in Cigarette Smoke Exposure and Influenza A Virus Infection-Induced Lung Injury

**DOI:** 10.1371/journal.pone.0123187

**Published:** 2015-05-01

**Authors:** Yashodhar P. Bhandary, Shwetha K. Shetty, Amarnath S. Marudamuthu, Krishna K. Midde, Hong-Long Ji, Homoyoun Shams, Renuka Subramaniam, Jian Fu, Steven Idell, Sreerama Shetty

**Affiliations:** 1 Texas Lung Injury Institute, Department of Medicine, University of Texas Health Science Center at Tyler, Tyler, Texas, United States of America; 2 Center for Research on Environmental Disease and Toxicology, College of Medicine, University of Kentucky, Lexington, Kentucky, United States of America; univeristy of alabama at birmingham, UNITED STATES

## Abstract

Parenchymal lung inflammation and airway and alveolar epithelial cell apoptosis are associated with cigarette smoke exposure (CSE), which contributes to chronic obstructive pulmonary disease (COPD). Epidemiological studies indicate that people exposed to chronic cigarette smoke with or without COPD are more susceptible to influenza A virus (IAV) infection. We found increased p53, PAI-1 and apoptosis in AECs, with accumulation of macrophages and neutrophils in the lungs of patients with COPD. In Wild-type (WT) mice with passive CSE (PCSE), p53 and PAI-1 expression and apoptosis were increased in AECs as was lung inflammation, while those lacking p53 or PAI-1 resisted AEC apoptosis and lung inflammation. Further, inhibition of p53-mediated induction of PAI-1 by treatment of WT mice with caveolin-1 scaffolding domain peptide (CSP) reduced PCSE-induced lung inflammation and reversed PCSE-induced suppression of eosinophil-associated RNase1 (EAR1). Competitive inhibition of the p53-PAI-1 mRNA interaction by expressing p53-binding 3’UTR sequences of PAI-1 mRNA likewise suppressed CS-induced PAI-1 and AEC apoptosis and restored EAR1 expression. Consistent with PCSE-induced lung injury, IAV infection increased p53, PAI-1 and apoptosis in AECs in association with pulmonary inflammation. Lung inflammation induced by PCSE was worsened by subsequent exposure to IAV. Mice lacking PAI-1 that were exposed to IAV showed minimal viral burden based on M2 antigen and hemagglutination analyses, whereas transgenic mice that overexpress PAI-1 without PCSE showed increased M2 antigen and inflammation after IAV infection. These observations indicate that increased PAI-1 expression promotes AEC apoptosis and exacerbates lung inflammation induced by IAV following PCSE.

## Introduction

Exposure to cigarette smoke (CS) is a common clinical problem associated with considerable morbidity, with an estimated 30% of cancer-related deaths attributed to tobacco use [1]. The United States Environmental Protection Agency has documented that each year there are 3000 lung cancer deaths and 53,000 coronary artery disease-related deaths in individuals with passive CS exposure (PCSE) [2]. PCSE induces airway injury and remodeling characteristic of chronic obstructive pulmonary disease (COPD), which is the fourth major cause of death in the United States [3]. The major components of extravascular fibrinolysis in lungs have been implicated in the pathogenesis of lung remodeling, participate in fibrotic repair and regulate airway and alveolar epithelial cell (AEC) viability. The pathophysiology of PCSE-induced lung inflammation has been directly linked to loss of alveolar architecture due to AEC apoptosis, suppressed body immunity and increased susceptibility to respiratory infections [4,5], which include infection with influenza A viral (IAV). PCSE/CSE can increase the morbidity of influenza infections, representing an important medical problem.

Plasminogen activator inhibitor-1 (PAI-1) is a serine protease inhibitor which irreversibly inhibits the activities of tissue-type plasminogen activator (tPA) and urokinase-type plasminogen activator (uPA). Defective alveolar fibrinolysis due to increased expression of PAI-1 is common in lung diseases such as acute lung inflammation(ALI), asthma, pneumonia, COPD, adult respiratory distress syndrome (ARDS) and interstitial lung diseases [6–13]. It has been reported that nicotine, a major constituent of CS, induces PAI-1 expression by endothelial cells and that plasma PAI-1 levels significantly increase in subjects who are exposed to CS [14,15]. We have recently reported that AECs exposed to PCS show augmented p53 and PAI-1 expression and that the p53-induced apoptosis of AECs is mediated by increased PAI-1 during bleomycin- or PCS- induced lung inflammation [16,17]. The process involves the binding of p53 through its C-terminal region to a 70 nucleotide *cis* element present in the 3’untranslated region of PAI-1 mRNA and stabilization of PAI-1 mRNA transcripts [18]. These observations lend support to the concept that PAI-1 plays a central role in PCSE induced lung inflammation.

However, little is now known about how PAI-1 affects the viability of AECs, alveolar inflammation or the severity of influenza A virus (IAV) infection after PCSE. In the present study, we demonstrate that a disproportionate increase in the expression of PAI-1 mediated by elevated p53 in AECs due to PCSE promotes lung inflammation. Further inhibition of PCSE-induced p53 and downstream PAI-1 mitigates pulmonary inflammation after PCSE followed by IAV-induced lung injury. Our study also shows that increased expression of PAI-1 aggravates the severity of IAV infection following PCSE. These observations link PCSE-induced alterations of PAI-1 to clinically important outcomes of IAV infection, including airway epithelial injury and lung inflammation associated with this infection.

## Materials and Methods

### Isolation of type II AECs from mouse lungs

Type II AECs were isolated according to the method described previously and the purity of the preparations was confirmed by lithium carbonate staining [19,20]. Type II AECs were maintained in AEC culture medium (ScienCell, CA). 293T cells from the American Type Culture Collection (Manassas, VA) and cultured in DMEM media containing 10% FBS.

### Construction of recombinant Adenoviral (Ad) vectors

A chimeric cDNA containing the SP-B 5’flanking sequences and 70 nt p53-binding PAI-1 mRNA 3’UTR sequences [18] was inserted into an empty Ad vector. A cDNA fragment that contains the SP-B 5’flanking sequences and sequences lacking p53-binding PAI-1 mRNA 3’UTR was also sub cloned into a promoter-less Ad vector and used as a control. These Ad vector constructs were transfected into 293T cells using Lipofectamine 2000 (Invitrogen) to obtain phage particles and viral titers measured as per the manufacturer’s protocol (Cell Biolabs, Inc). The Ad vector containing the SP-B 5’flanking DNA linked to p53-binding or control non-p53-binding 3’UTR sequences were exposed to type II AECs as described elsewhere [21].

### Mice

WT, p53- and PAI-1-deficient mice as well as transgenic mice that overexpress PAI-1 of C57BL-6 background were purchased from the Jackson Lab (Bar Harbor, ME), bred at The University of Texas Health Science Center at Tyler.

### Human tissue samples

Human lung tissues were obtained from Lung Tissue Research Consortium (LTRC) and from The Department of Pathology; The University of Texas Health Science Center at Tyler (UTHSCT) using IRB approved exempt protocol [16, 22] and was deidentified prior to analysis. Lung tissues from patients with a clinical diagnosis of COPD were obtained. The clinical diagnosis of the patients is generally established based on documented airways obstruction by spirometry, a history of cigarette smoking exposure and a compatible clinical picture. These specimens were usually obtained from patients undergoing resections for lung cancer superimposed on clinical COPD. Both active smokers and those with a past history of smoking were included in this cohort. Normal lung tissues were histologically normal tissues typically obtained from patients undergoing lung resections of neoplasms, with the surrounding normal lung committed to these studies. These patients did not have a clinical diagnosis of COPD nor histologic evidence of emphysema or chronic bronchitis.

### Preparation of CS extract

CS extracts were prepared using research cigarettes 2R4F from the Tobacco Health Research University of Kentucky (Lexington, KY) by following the method developed by Carp and Janoff [23]. One cigarette at each interval was burned in a side arm flask and the smoke generated was bubbled into phosphate buffered saline at room temperature (22°C) through an attached peristaltic pump. Enough cigarettes were burned to reach an absorbance of 1.0 at 230 nm, which is considered 100%. CS extract was filter sterilized by passing it through a 0.2 μm filter. Fresh CS was prepared to desired concentrations in serum free media and used within 30 minutes.

### PCSE

All experiments involving mice were performed according to the approved protocols under the guidelines of Animal Care and Use Committee of The University of Texas Health Science Center at Tyler (**Permit Number:** A3589-01) [16,17]. WT, p53^-/-^ and PAI-1^-/-^ mice were exposed to PCS from 40 cigarettes over a 2h period five days per week for 20 weeks (~90 mg/m^3^ total solid particulates) using a mechanical smoking chamber (Teague Enterprises, Davis, CA) as we described [17]. Control mice remained in ambient air in otherwise identical conditions. Mice were euthanized by IP injection of Euthasol 5 μl/g body weight after which their lungs were harvested and used for various analyses.

### Influenza A virus infection

Mouse-adapted influenza virus A/Puerto Rico/8/34 (PR8) (H1N1) strain was used in all experiments. For IAV infection, WT, PAI-1^-/-^ and PAI-1^+/+^ mice were intranasally inoculated with 50 μl of PBS containing purified H1N1 PR8 stain (0.5 LD50) under light general anesthesia with a combination of Ketamine/Xylazine [24]. All infected mice were monitored for weight loss and mortality on a daily basis. For IAV infection post PCSE, WT and PAI-1-deficient mice were exposed to PCS for 19 weeks and were later treated with saline or purified IAV as described above. Control mice in ambient air were also exposed to 50 μl saline through intranasal instillation. One week after IAV infection, mice were euthanized and lungs were harvested for various analysis. For hemagglutination analyses, lungs from WT and PAI mice were homogenized and diluted ten-fold before exposure to Madin-Darby Canine Kidney (MDCK) cells in 96-well culture dishes. These cells were cultured in RPMI 1640 (Life technologies, CA) supplemented with 10% FBS at 37°C and 5% CO2. After 72h, 50μl of culture medium from infected MDCK cells added to 50μl of 0.5% chicken RBCs in 96-well round bottom plates, swirled and incubated at room temperature. Hemagglutination or the lack thereof was recorded after an hour and the tissue culture infective dose (TCID50) of IAV was calculated by the Spearman-Karber formula.

### Western blotting of AEC lysates

Lung homogenates or isolated Type II AEC lysates were subjected to SDS-PAGE, transferred to nitrocellulose membrane and incubated with primary antibodies at 1:1000 dilutions at 4°C overnight followed by reaction with goat anti-rabbit or anti-mouse-HRP-conjugated secondary antibody at 1:1000 dilutions for 1 h at room temperature. Protein bands were visualized by enhanced chemiluminescence detection method as we described earlier [16–18,21,25].

### Measurement of myeloperoxidase (MPO) activity

Mouse lung MPO activity was determined as described previously [26,27]. Briefly, lungs were suspended in 1 ml buffer (0.5% hexadecyl-trimethylammonium bromide in 50 mM phosphate buffer, pH 6.0) and sonicated at 30 cycles twice for 30 seconds on ice. Homogenates were centrifuged at 12,000 rpm and MPO activity in supernatants measured by colorimetric method using *o*-dianisidine dihydrochloride and H_2_O_2_ in a 96-well plate. MPO activity levels in lung samples were determined from a standard curve generated using known amounts of purified MPO (Sigma, MO).

### Immunohistochemical (IHC) and immunofluorescence assay

Lung sections (5 μm) were subjected to IHC analysis using antigen detection kit provided by Lab Vision (Fremont, CA) as we described elsewhere [16,17,21]. Lung sections exposed to rabbit IgG served as negative controls. The numbers of neutrophils and macrophages were assessed via immunohistochemical staining of lung sections by counting positive cells in 10 high-powered fields (hpf, original magnification×400) per section. For immunofluorescence assays, lungs sections were incubated overnight with primary antibody or control IgG. These sections were later treated with fluorochrome conjugated secondary antibody. The lung sections were then examined by fluorescent microscopy.

### RT-PCR

Total RNA was isolated from lung tissues or isolated type II AECs using TRI reagent and reverse transcribed using impromII Reverse transcription kit (Promega, WI USA). For PCR, EAR (forward primer: 5’GATCGAATTCAATACTTTTCTTCATACAA-3’ and reverse primer: 5’GATCGGATCCGTGAACTGGAACCACTGGATA-3’ and β-actin (forward: 5’CACCGCAGCTCGTAGCTCTTCTCCAGGG-3’ and reverse: 5’CCAGCCATGTACG TTGCTATCCAG-3’ primers were used to amplify aliquots of cDNA respectively [28]. The thermal cycling profile for EAR and β-actin was 94°C for 2 minutes, followed by 35 cycles of 94°C for 1 minute, 63°C for 1 minute, and 72°C for 1 minute, with a final extension of 72°C for 2 minutes.

### Assessments of lung epithelial cell apoptosis

To study the programmed cell death in the lungs of PCSE-treated mice, we used the terminal uridine deoxynucleotidyl transferase (dUTP) nick end-labeling (TUNEL) staining technique. The lungs from each animal were sectioned and were blocked with hydrogen peroxidase, incubated with biotinylated nucleotides in the presence of terminal deoxynucleotidyl transferase (TdT) enzyme for 1 hour at 37°C. The sections were then incubated with horse radish peroxidase (HRP) substrate and diaminobenzidine (DAB) chromogen to label the nicked DNA. Large conducting airways, small airways including terminal bronchioles and respiratory bronchioles, and alveoli were examined by a lung pathologist (TCA). The entire lung section of each case was examined sequentially at high power (400x). The large conducting airways, small airways, and alveolar cells from the hpf were scored for strong brown nuclear staining within epithelial cells indicating TUNEL positivity. Cells were scored as follows: (1) average of 0 to 2 positive nuclei per 10 hpf, (2) average of 3 to 6 positive nuclei per 10 hpf, (3) average of 7 to 10 positive nuclei per 10 hpf, (4) average of 11 to 20 positive nuclei per 10 hpf and (5) average of 21 or more positive nuclei per 10 hpf. The large airways, small airways, and alveoli were also evaluated for airway remodeling, including smooth muscle hyperplasia and sub mucosal airway fibrosis, as well as for macrophage and other inflammatory cell infiltration, and emphysematous change.

### Statistical analysis

Statistical significance between two groups was analyzed by Student’s *t* test and for multiple groups by two-way ANOVA tests. Graph pad Prism 4.0 software was used to analyze statistical differences.

## Results

**Fig 1:**
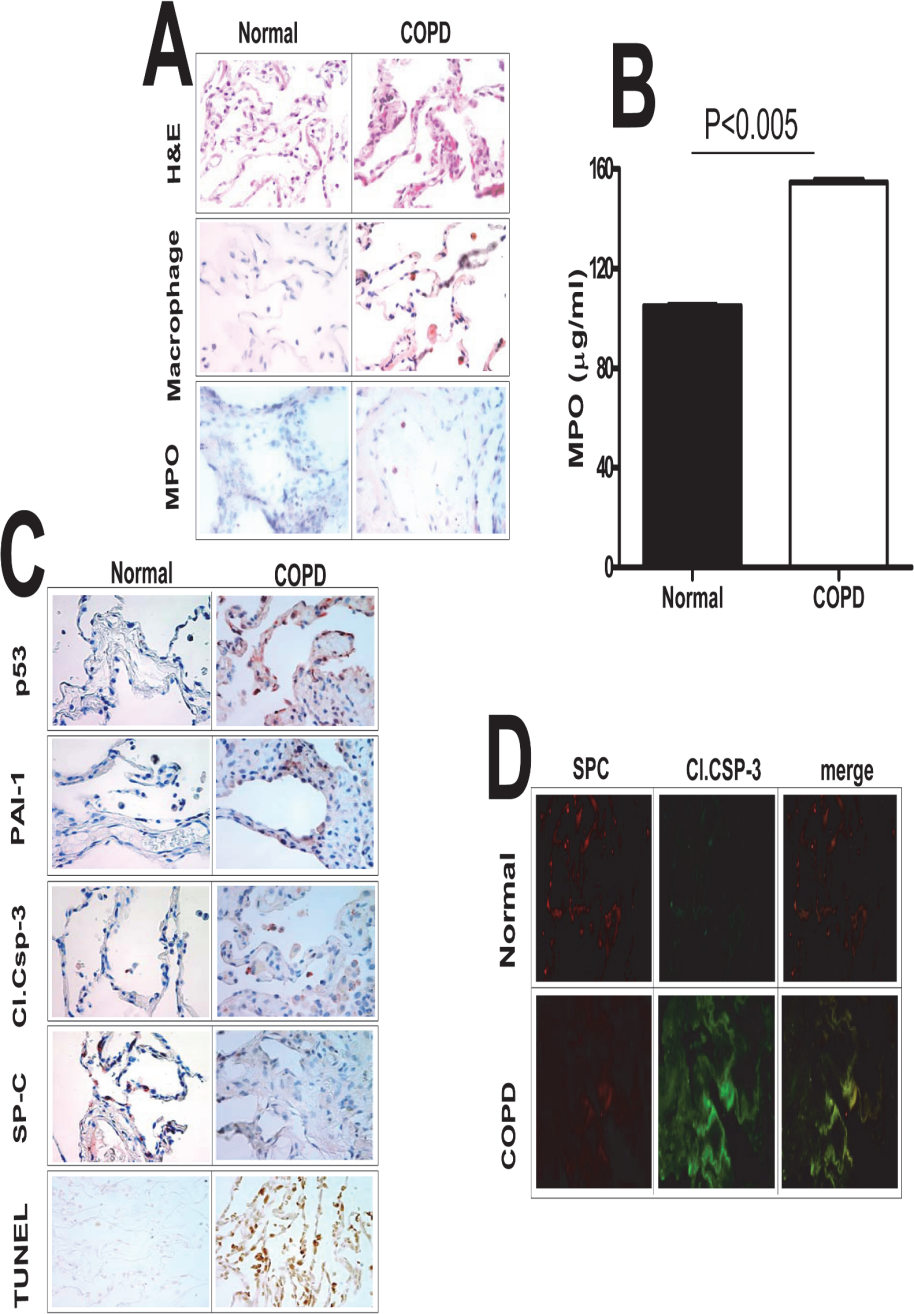
Increased p53 and PAI-1 antigen levels, and AEC apoptosis in the lung tissues of patients with COPD. (**A**) Paraffin embedded sections from COPD and histologically “normal” donor lung tissues were subjected to H & E and IHC staining for macrophages and myeloperoxidase (MPO) using specific antibodies. (**B**) Lung homogenates from COPD (n = 3) and histologically “normal” donor lung tissues from control patients were also tested for MPO activity by colorimetric assay. Data shown in bar graphs are mean ± SD of two independent experiments. (**C**) The lung sections were also subjected to IHC analysis using anti-p53, anti-PAI-1, anti-active caspase-3 and anti-SP-C antibodies to assess their expression and apoptosis in AECs. Lung sections were also subjected to TUNEL staining to assess apoptosis. (**D**) Immunofluorescence staining was performed for the above lung sections using anti-SP-C and anti-active caspase-3 primary antibodies and fluorescently labeled secondary antibodies to assess apoptosis of type II AECs. Representative fields from 1 of 3 sections per subject are shown at X 400 magnification.

We previously reported that p53 induces PAI-1 through inhibition of PAI-1 mRNA degradation and we also demonstrated that inhibition of PCSE-induced p53 or PAI-1 prevents AEC apoptosis in murine lungs [17,18]. Therefore, we sought to extend this work and initially determine the impact of this system on human COPD. We first analyzed lung sections of patients with COPD to characterize the injury and the responses were compared with control patients without COPD. Hematoxylin and Eosin (H&E) staining of COPD lung sections revealed increased pulmonary inflammation. Further, differential staining for macrophage and MPO activity confirmed increased accumulation of both macrophages and neutrophils in COPD lungs (Fig 1A). Analysis of lung homogenates for MPO activity confirmed increased neutrophil accumulation in COPD lungs (Fig 1B). IHC analysis of COPD lung sections also showed increased AEC apoptosis associated with induction of p53 and PAI-1 and reduced SP-C expression (Fig 1C). Immunofluorescence and co-localization (Fig 1D) indicated that apoptosis was predominantly restricted to type II AECs, indicating a temporal, spatial and cell-specific link between increased p53 and PAI-1 expression and apoptosis in type II AECs of the lungs of COPD patients.

**Fig 2:**
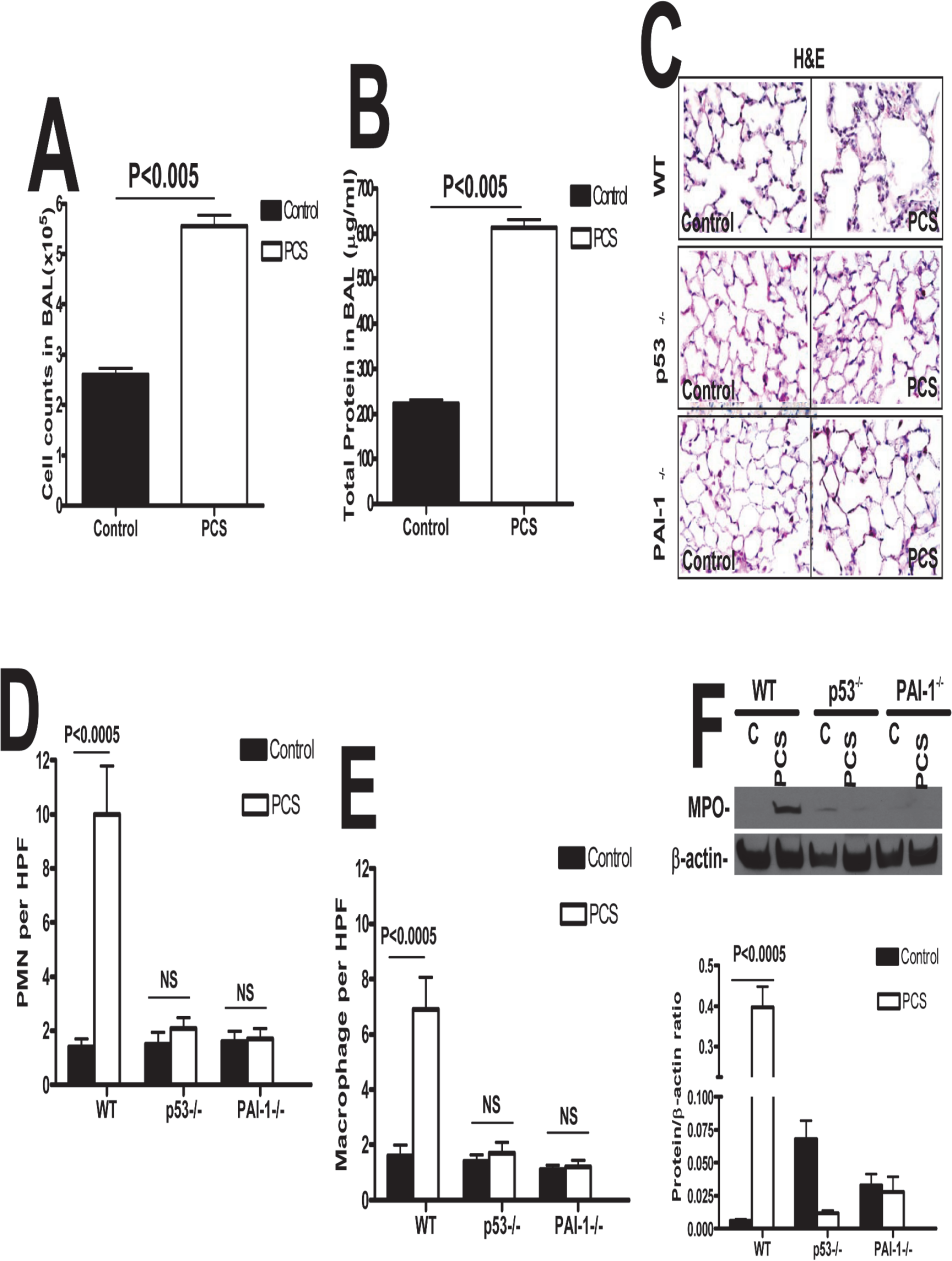
p53-mediated induction of PAI-1 expression contributes to increased pulmonary MPO levels in mice with PCSE. (**A**) WT mice were exposed to ambient air (control) or PCS (n = 5/group) for 20 weeks. LL collected from these mice was subjected to total cell counting. (**B**) Total protein in the lavage was quantified from the above exposed mice. (**C**) Paraffin embedded lung sections from WT, p53- and PAI-1-deficient mice (n = 5mice/group) exposed to ambient air (control) or PCS for 20 weeks were subjected to H&E staining. Representative fields from 1 of 3 sections per subject are shown at X 400 magnification. Lung sections were subjected to IHC analysis for neutrophils using anti-MPO antibody and for macrophages using anti-F4/80 antibody. Neutrophils (**D**) and macrophages (**E**) were counted in 10 high-power fields (hpf) are shown as bar graph. (**F**) Lung homogenate from WT, p53- and PAI-1-deficient mice exposed to ambient air or PCS for 20 weeks were immunoblotted for changes in the levels of MPO using anti-MPO antibody. These membranes were later stripped and analyzed for β-actin to assess loading. Data shown in bar graphs are mean ± SD of two independent experiments (n = 5 mice/group). Differences between treatments are statistically significant *(P<0.05).

p53 induces PAI-1 expression during PCSE-induced lung inflammation[17,18]. To confirm the inferred involvement of p53-mediated induction of PAI-1 in PCSE-induced lung inflammation, we analyzed the lung lavage (LL) fluids from the WT mice exposed to PCS. LL total cell counts and protein indicated a significant increase in total number of leukocytes and protein contents compared to ambient air exposed controls (p<0.005, Fig 2A & 2B). We next exposed the WT, p53- and PAI-1- deficient mice to PCS. The dosage (90 mg/m^3^ total particulate matter) and duration (20 weeks) were selected based on the reported literature by many laboratories including ours [17] for second hand CS exposure. The total particulate matter of 90 mg/m^3^ is within the concentration limits reported by other laboratories [29–31]. Earlier report also showed that exposure of mice to higher dose (>250–300 mg/m^3^ total particulate matter) of CS for extended period (9 months or more) might develop discernible emphysema [32]. Since p53-deficient mice spontaneously develop lymphoma as they age beyond 5 months and our main focus was to assess the contribution of p53 and PAI-1 in cigarette smoke induced lung epithelial cell apoptosis, we limited the animals to 20 weeks of exposure. We next stained lung sections of mice exposed to PCS for 20 weeks with H & E. Lung sections of WT mice exposed to PCS showed increased accumulation of leukocytes compared to control mice exposed to ambient air (Fig 2C). However, mice lacking either p53 or PAI-1 resisted PCSE-induced lung inflammation. Further quantitative analysis of neutrophils and macrophages through IHC analysis for MPO and macrophages antigens confirmed an increased influx of neutrophils (Fig 2D) and macrophages (Fig 2E) in WT PCSE mice, which were significantly reduced in mice lacking p53 or PAI-1 expression. Analysis of lung homogenates showed increased MPO activity in WT mice exposed to 20 weeks of PCS (Fig 2F), which was significantly reduced in both p53- and PAI-1-deficient mice.

**Fig 3:**
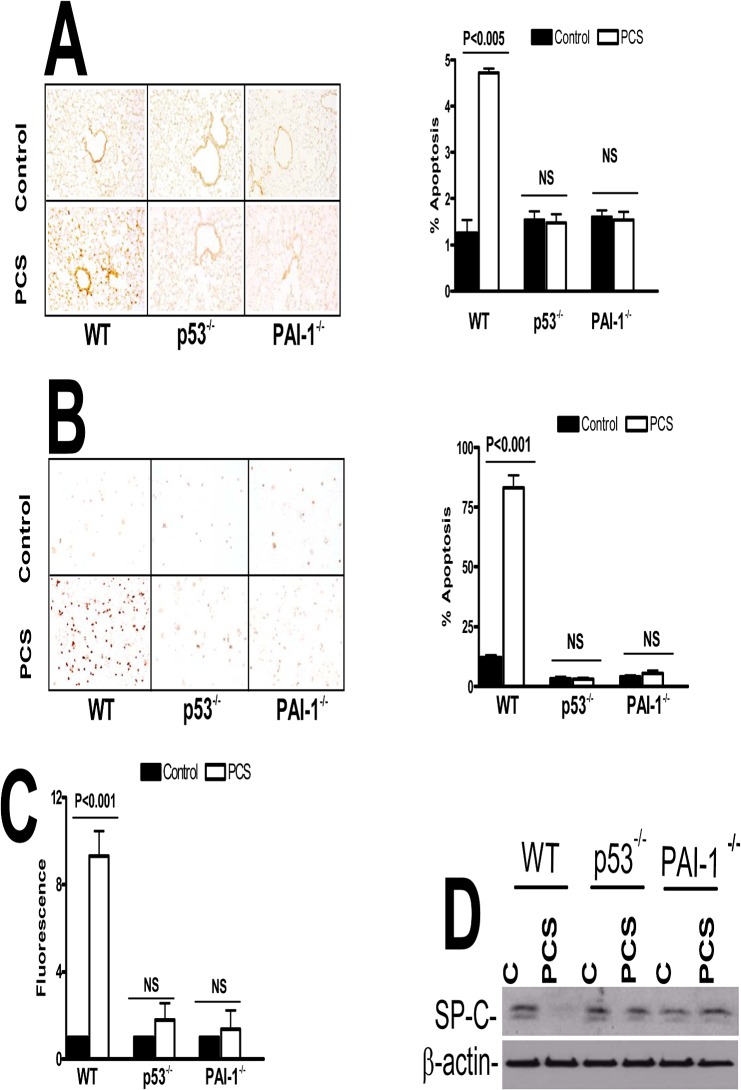
p53 and PAI-1 are prominently linked to PCSE-induced type II AEC apoptosis. (**A**) WT, p53- and PAI-1-deficient mice were exposed to ambient air (control) or PCS for 20 weeks. Lung section obtained from these mice were subjected to TUNEL staining and the bar graph represents percent apoptosis in these groups with error bars and significance *(p<0.005) (n = 5 mice/group). (**B**) Type II AECs isolated from WT, p53- and PAI-1-deficient mice as described in methods were subjected to TUNEL staining and the bar graph represents percent apoptosis in these groups with error bars and significance *(p<0.005) (n = 5 mice/group). (**C**) Type II AECs isolated from WT, p53- and PAI-1-deficient mice were subjected to flow cytometric analysis after staining with anti-annexin-v antibody and PI to assess apoptosis. NS = the differences are not statistically significant (n = 5 mice/group). (**D**) Type II AECs isolated from WT and p53- and PAI-1-deficient mice as described above were immunoblotted for SP-C and β-actin as a loading control.

Since p53-mediated induction of PAI-1 contributes to increased lung inflammation during PCSE-induced lung injury, lung sections from control and PCS-treated mice were next stained for nicked DNA by TUNEL analyses to assess AEC apoptosis. WT, PAI-1^-/-^, and p53^-/-^ non-smoked cases averaged slightly more background apoptotic scores in the conducting airways (1.4, 1.4, and 1.8, respectively) than in the small airways (1.0, 1.0, and 1.0 respectively) or the alveoli (1.0, 1.0, and 1.0, respectively). The PAI-1^-/-^ and p53^-/-^ smoked mice averages were similar to the ambient-air exposed control mice (1.6 and 1.4 for conducting airways, 1.0 and 1.0, respectively, for both small airways and alveoli). The WT PCS-treated mice uniformly demonstrated higher apoptotic scores as compared to WT control cases, and compared to PAI-1^-/-^ and p53^-/-^ PCS-treated mice for all areas-conducting airways (5.0, 1.6, and 1.4, respectively), small airways (4.2, 1.0, and 1.0, respectively), and alveoli (4.8, 1.0, and 1.0, respectively). Fig 3A represents findings that were uniformly demonstrated in each animal of each group. We found a relative paucity of AEC apoptosis in control WT, PAI-1- and p53-deficient control mice that were not exposed to PCS and the relatively strong TUNEL-positivity in the airway epithelium of WT PCSE mice. The data reveals that the AEC apoptosis, considered as an average of airway, small airway and alveolar findings in each animal, was significantly increased in the lungs of WT mice exposed to PCS (p<0.005) (Fig 3A).

We next isolated type II AECs from both control and PCS mice and lithium carbonate staining for inclusion bodies confirmed that the purity of the preparations was >95%. The percentage of TUNEL positivity within type II AEC averaged 6.4, 4.12, and 5.4% in the WT, PAI-1^-/-^, and p53^-/-^ control cases respectively and averaged 74.2, 6.06, and 5.4% in the WT, PAI-1^-/-^ and p53^-/-^ animals (n = 5/group) exposed to PCS, respectively. Fig 3B illustrates the relatively negative staining observed in the control ambient air-exposed WT, p53^-/-^ or PAI-1^-/-^ mice and PCS-exposed PAI-1^-/-^ or p53^-/-^ mice. Striking positivity occurred in the nuclei of AECs from each of the PCS-exposed WT mice. These findings are represented in a bar graph in which data from each mouse is illustrated, showing the uniformity of the responses in each group (n = 5/group, P<0.001). Analysis by flow cytometry after annexin and propidium iodide (PI) staining further confirmed more type II AEC apoptosis in WT mice with PCSE (Fig 3C). Both p53- and PAI-1-deficient mice resisted AEC apoptosis. This is supported by decreased SP-C expression in WT mice exposed PCS when compared to control WT mice or those lacking p53 or PAI-1 expression (Fig 3D).

**Fig 4:**
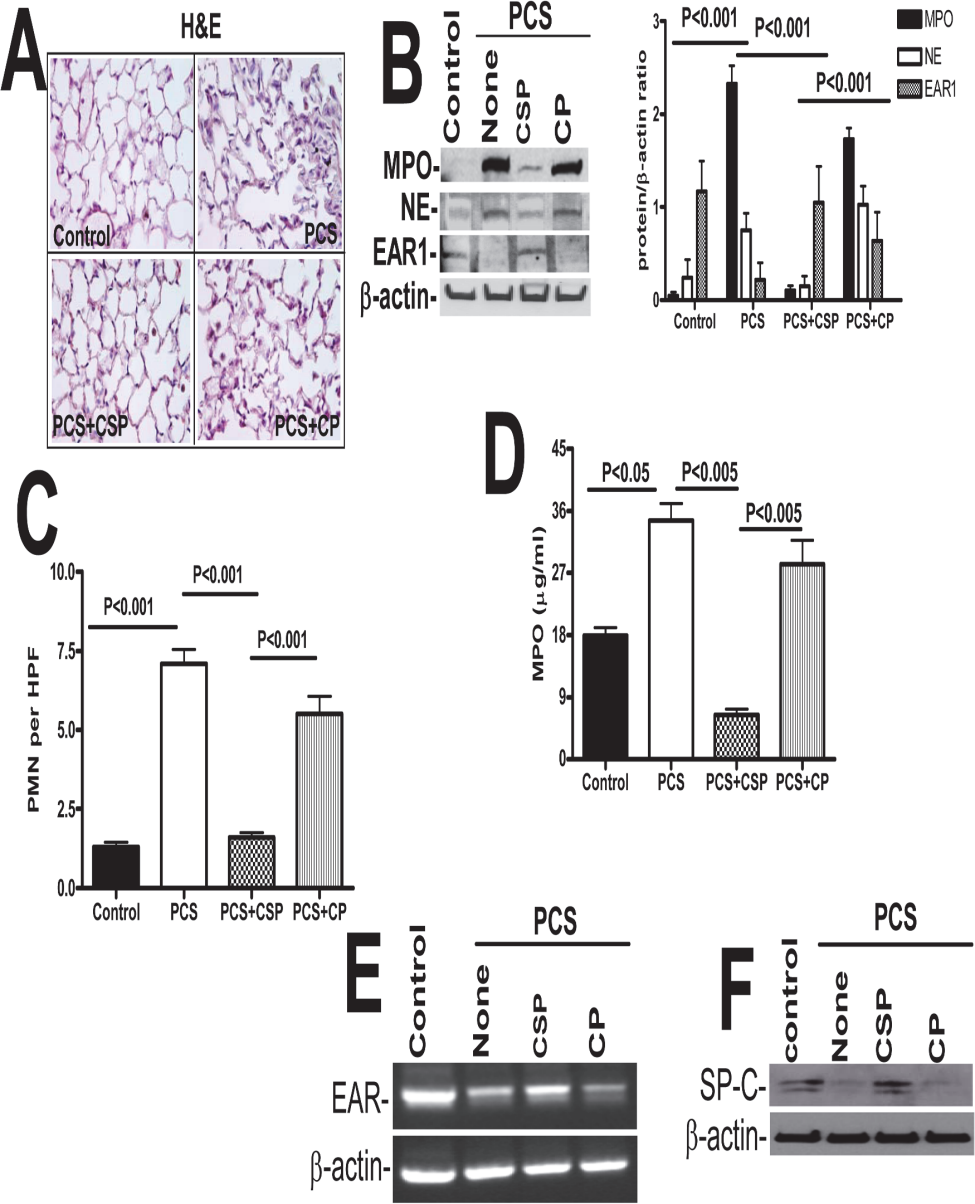
CSP inhibits PCSE-induced induced MPO and neutrophil elastase in mice. WT mice were exposed to ambient air or PCS as described in the Methods for 5 days per week. After 4 weeks of PCS exposure, mice exposed to PCS were IP injected with or without 18.75 mg/kg body weight of CSP or CP once every week for 4 more weeks. After 20 weeks of PCS exposure, mice were euthanized. (**A**) Paraffin embedded lung sections from WT mice were subjected to H & E staining. Representative fields from 1 of 3 sections per subject are shown at X 400 magnification. (**B**) Lung homogenates from these mice were tested for changes in MPO, neutrophil elastase (NE), EAR1 and β-actin by Western blotting. Densities of individual bands normalized against β-actin are shown in a bar graph of two independent experiments (n = 5 mice/group). (**C**) Lung sections of the mice were subjected to IHC analysis using anti-MPO antibodies. Neutrophils were quantified by counting positive cells in 10 high-power fields (hpf) are shown as bar graph. (**D**) Lung homogenates from WT mice exposed to ambient air or PCS treated with or without CSP or CP were tested for MPO activity by colorimetric assay. (**E**) Total RNA obtained from the lungs of these mice were tested for changes in the expression of EAR and β-actin mRNA by RT-PCR. Experiments were repeated at least two times (n = 5 mice/group). (**F**) Type II AECs isolated from the mice as described above were immunoblotted for SP-C with β-actin as the loading control.

Mice lacking either expression of p53 or PAI-1 resist PCSE-induced lung inflammation, which is otherwise increased in WT mice. We found that inhibition of PCSE-mediated induction of p53 expression and downstream PAI-1 by p53 using caveolin-1 scaffolding peptide (CSP) [16] significantly suppressed lung inflammation in WT mice. CSP peptide is a stretch of amino acid residues located in the N-terminus caveolin-1 protein, which has been shown to serve as a structural scaffold responsible for interactions with many proteins, including enzymes involved in cellular signaling [16,17]. On the other hand, those that received a control peptide (CP) of scrambled sequence showed increased inflammation (Fig 4A). Western blot analysis for MPO and neutrophil elastase showed that CSP decreased their expression when compared to CP treated mice exposed to PCS. Next we wanted to check EAR1 level in mice exposed to PCS. EAR1 is an epithelial-derived innate immune protein expressed by type II AECs, eosinophils, neutrophils and macrophages that may limit viral growth during the early stages of an infection. Treatment of WT mice with CSP reversed PCSE-induced suppression of EAR1, while in those exposed to CP after PCSE-injury still showed reduced EAR1 expression (Fig 4B). Quantitation of neutrophils through IHC analysis of lung sections for MPO antigens showed increased neutrophil levels in PCS exposed mice, however CSP treated mice showed reduction in neutrophils compared to those treated with CP (Fig 4C). Further colorimetric analysis of lung homogenates for MPO activity (Fig 4D) indicated that CSP and not CP reduced PCSE-mediated accumulation of neutrophils. Since CSP restored EAR1 protein which is otherwise reduced after PCSE-induced lung injury, we next analyzed the EAR mRNA levels in those mice. We found that EAR mRNA levels were decreased in these mice; while CSP but not CP treated mice reversed the EAR mRNA levels (Fig 4E). Western blotting of lung homogenates indicated that CSP restored SP-C expression in mice exposed to PCS when compared to those with PCSE alone or PCSE plus CP (Fig 4F) indicating protection of AECs against apoptosis.

**Fig 5:**
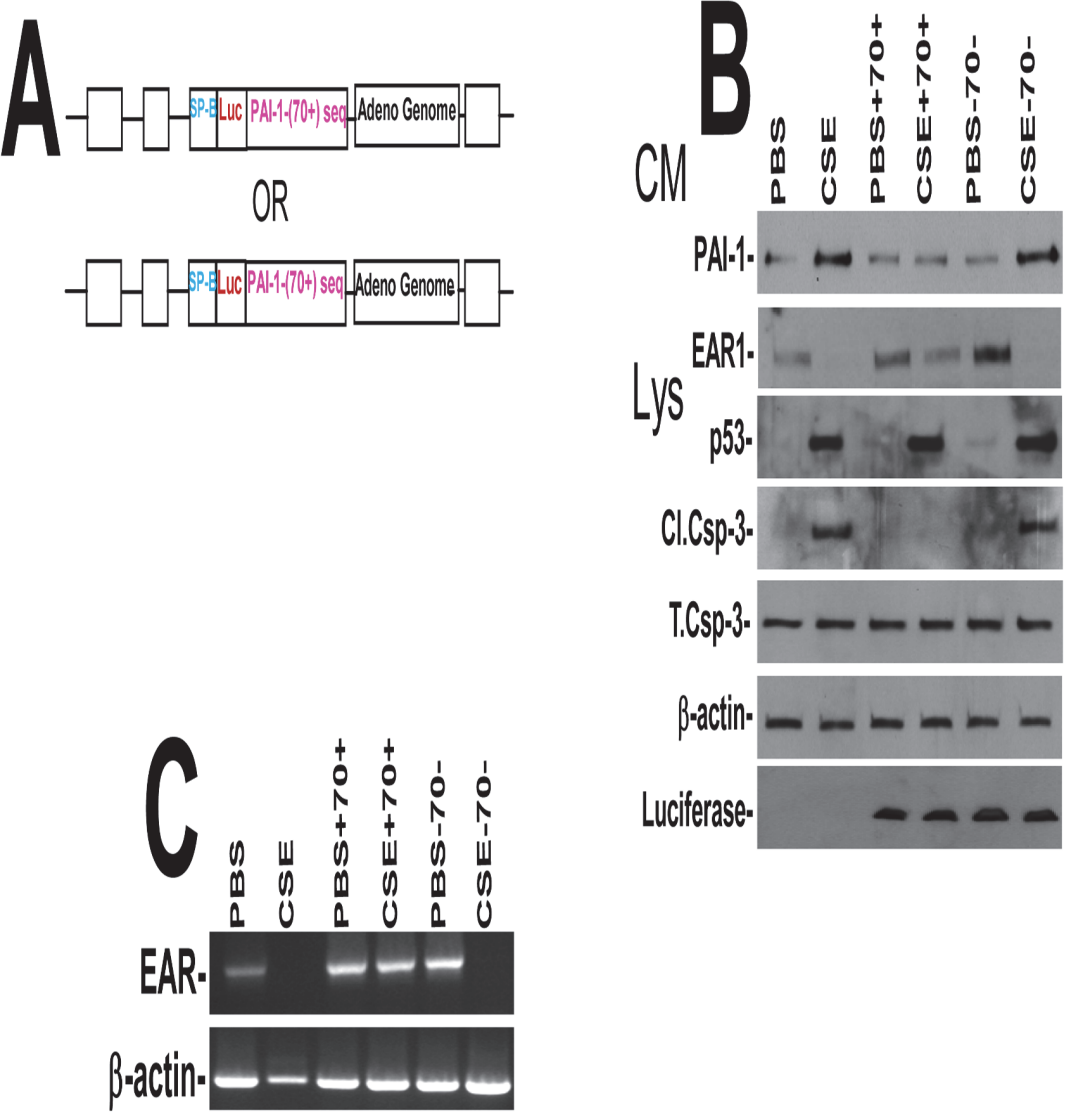
(A) Map showing the Ad-vector harboring SP-B promoter plus chimeric luciferase cDNA having either p53-binding (Ad-PAI-1^70+^) or corresponding control (Ad-PAI-1^70-^) PAI-1 3’UTR sequence. (**B**) Type II AECs isolated from WT mice were transduced with Ad-vector alone or Ad-PAI-1^**70+**^ or Ad-PAI-1^**70-**^ *in vitro*. One day after transduction, these cells were either treated with PBS or 1.5% of CS extract (CSE, O.D. = 1.00 at 260 nm = 100%) for additional 24h. Conditioned media (CM) were tested for changes in PAI-1 and EAR1, and the cell lysates (CL) were immunoblotted for p53, luciferase and active caspase-3. (**C**) Total RNA obtained from WT AECs as described above was analyzed for EAR mRNA by RT-PCR. Experiments were repeated at least two times.

To confirm the specificity of p53-mediated induction of PAI-1 in CS-induced AEC injury, we blocked CS-induced p53 from binding to PAI-1 mRNA by expressing Ad vector expressing p53-binding 3’UTR sequence (Fig 5A), which competes with endogenous PAI-1 mRNA for p53 [18,21]. We found that expression of p53-binding 3’UTR sequences, but not control sequences, significantly suppressed CS-induced PAI-1 expression and apoptosis without affecting p53 expression in AECs (Fig 5B). These were otherwise increased after CS induced injury. Further, EAR1 protein and EAR mRNA expression by AECs were also reduced after CS exposure. These changes were reversed by forced expression of p53-binding 3’UTR but not control sequences (Fig 5C).

**Fig 6:**
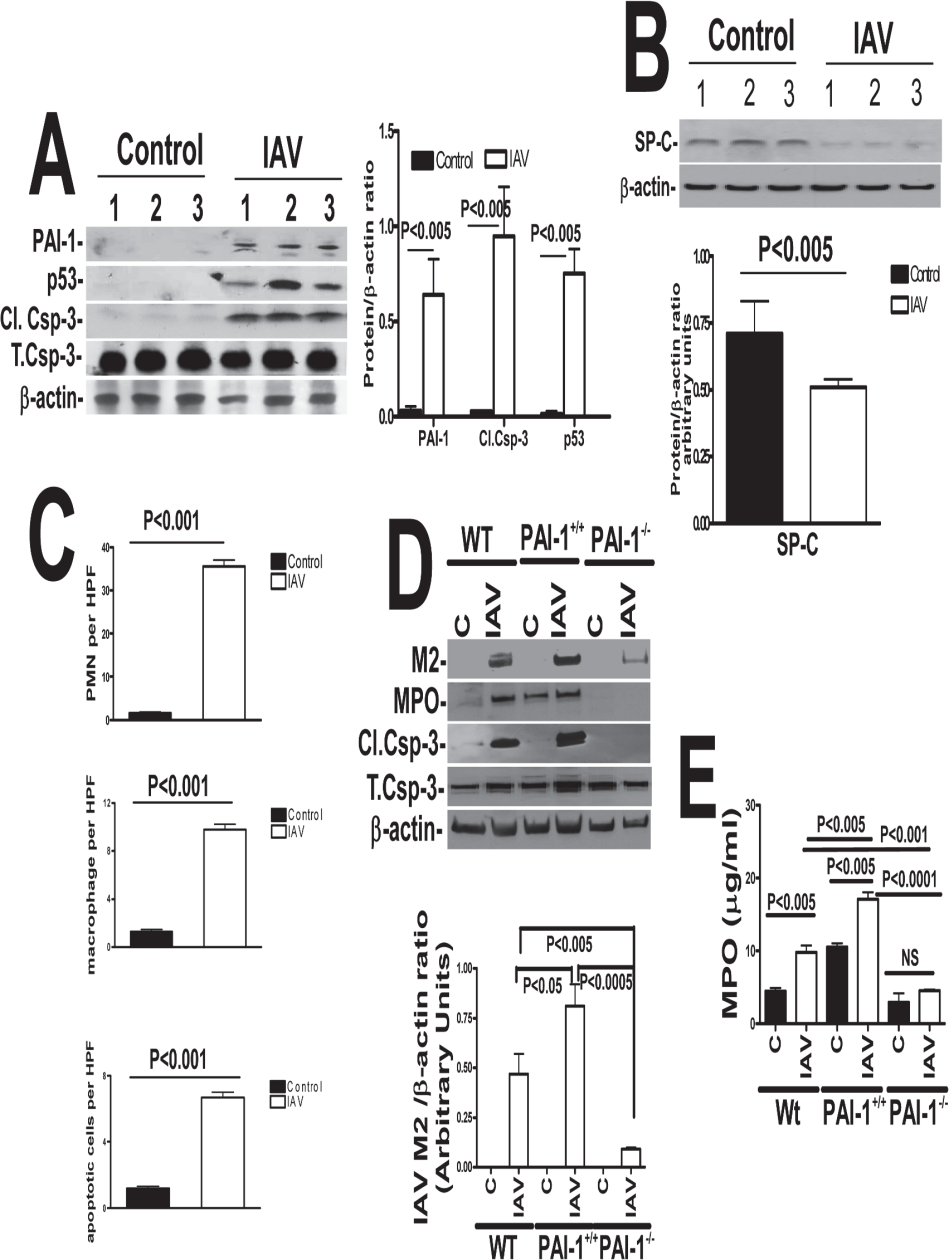
Increased PAI-1 expression sensitizes mice to IAV infection and alveolar injury. (**A**) Mice (n = 3) treated with saline or 0.5 LD_50_ of purified mouse-adapted IAV (strain A/PR/8/34) in 50 μl by intranasal instillation. LL fluids were analyzed for PAI-1, and isolated type II AECs lysates were immunoblotted for p53, activation of caspase-3 and β-actin. Data shown in bar graphs are means ± SD of two independent experiments. Differences between treatments are statistically significant *(P<0.05) (n = 3 mice/group). (**B**) Mice exposed to saline or IAV as described above were analyzed for SP-C and β-actin. Data shown in bar graphs are means ± SD of two independent experiments. Differences between treatments are statistically significant *(P<0.05) (n = 3 mice/group). (**C**) Lung sections from the mice treated as described above were subjected to IHC analysis for MPO and macrophage antigens, and TUNEL staining to assess inflammation and apoptosis. Neutrophils, macrophages and apoptotic (TUNEL-positive) cells were quantified by counting positive cells in 10 high-powered fields (hpf) are shown as bar graph. (**D**) WT mice or transgenic mice that over express PAI-1 (PAI-1^+/+^) or PAI-1-deficient mice (PAI-1^-/-^) were exposed to 50 μl saline or IAV in saline. Lung homogenates were immunoblotted for changes in IAV M2, MPO and active caspase-3 antigen levels to assess severity of IAV infection, inflammation and lung injury. β-actin was tested to gauge similar loading. Bar represents fold changes in the densities of bands (IAV M2) normalized against β-actin levels in the same sample (n = 3 mice/group). (**E**) Lung homogenates from WT mice or transgenic mice that overexpress PAI-1 (PAI-1^+/+^) or PAI-1-deficient mice (PAI-1^-/-^) were also tested for MPO activity by colorimetric assay. Data shown in bar graphs are means ± SD of two independent experiments (n = 3 mice/group).

Because epidemiological studies revealed that people with PCSE are several fold more likely to incur IAV infection than non-exposed subjects [33], we next sought to identify mechanisms by which PCSE increases susceptibility to IAV-induced injury. We found that WT mice exposed to IAV showed increased p53 and PAI-1 in AECs associated with a parallel increase in active caspase-3 (Fig 6A), indicating that IAV, like PCSE-induced lung inflammation augments AEC apoptosis. Further analysis of lung homogenates by western blotting indicated that IAV infection significantly reduced SP-C expression (Fig 6B). Quantitative analysis of lung sections of mice exposed to IAV for MPO and macrophage antigens and TUNEL staining indicated increased neutrophil and macrophage accumulation in the lung tissues with induction of AEC apoptosis (Fig 6C). We next exposed WT mice to IAV and analyzed M2 antigens in lung homogenates to assess viral load. The responses were compared with mice lacking PAI-1 expression or transgenic mice that overexpress PAI-1. As shown in Fig 6D, lung homogenates of WT mice as well as transgenic mice that overexpress PAI-1 expressed M2 antigens indicating IAV infection following intranasal exposure to IAV. Interestingly, homogenates of mice lacking PAI-1 expression and exposed to IAV showed relatively minimal M2 antigen expression compared with corresponding expression levels in WT or transgenic mice. This suggests that increased PAI-1 expression may foster an increased severity of IAV infection. Similarly, analyses of lung homogenates of WT and transgenic mice that overexpress PAI-1 indicated augmented epithelial apoptosis following IAV infection, while those lacking PAI-1 expression resisted activation of caspase-3. MPO activity in the lung homogenates of WT mice exposed to IAV increased, commensurate with increased inflammation (Fig 6E). In the case of transgenic mice that overexpress PAI-1, baseline levels of MPO activity was much higher than that of WT mice. Transgenic PAI-1-overexpressing mice exposed to IAV also showed significantly more MPO activity in the lungs than corresponding levels in WT mice, while PAI-1-deficient mice had relatively decreased IAV-induced pulmonary MPO activity. This indicates that increased PAI-1 expression contributes to inflammation associated with IAV infection.

**Fig 7:**
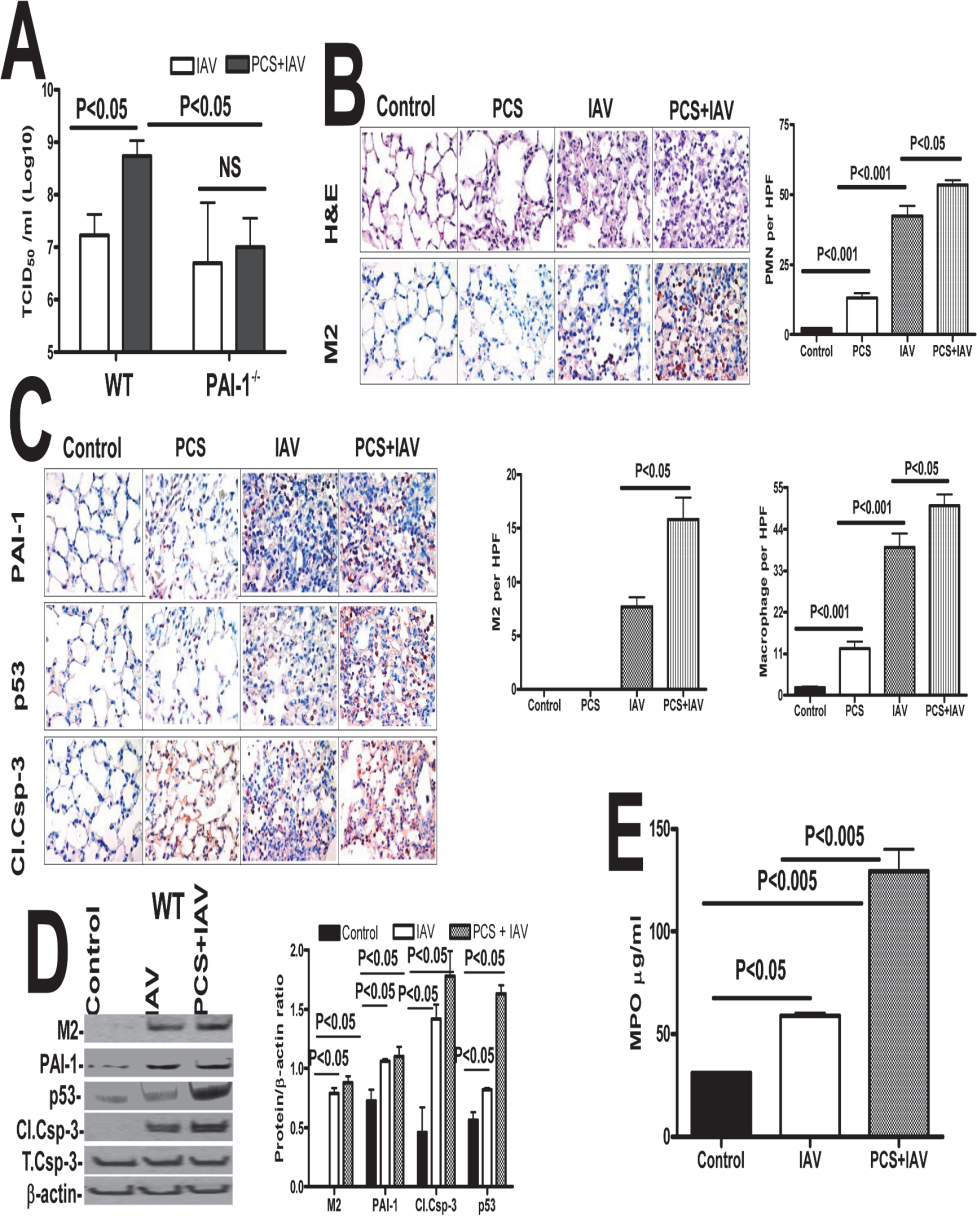
Increased IAV infection in mice with PCSE is associated with augmented p53 and PAI-1 expression, and type II AEC apoptosis. **(A)** Mice exposed to ambient air (AIR) or PCS were treated with 50 μl saline or IAV in saline via intranasal instillation. One week after IAV infection, these mice were euthanized. Lungs homogenates were quantified for viral titers using hemagglutination assay. **(B)** Mice exposed to ambient air (AIR) or PCS were treated with 50 μl saline or IAV in saline via intranasal instillation. One week after IAV infection, these mice were sacrificed. Sections (5 μM) from the inflated lungs were subjected to H & E staining, IHC analysis to detect viral protein M2, neutrophil and macrophage staining using specific antibodies. Representative fields from 1 of 3 sections per subject are shown at X 400 magnification (n = 5 mice/group). The changes in neutrophils, macrophages and M2 levels in the lung sections were quantified by counting positive cells in 10 high-powered fields (hpf) are shown as bar graph. (**C**) Mice exposed to ambient air or PCS for 19 weeks were treated with 50 μl saline or 0.5 LD50 of purified IAV in saline through intranasal instillation. One week after IAV infection these mice were sacrificed. Lung sections were analyzed for changes in p53 and PAI-1 and active caspase-3 antigen levels by IHC. Representative fields from 1 of 3 sections per subject are shown at X 400 magnification. (**D**) Lung homogenates were immunoblotted for changes in IAV M2 antigens to assess severity of IAV infection and also for changes in p53 and PAI-1 expression and active caspase-3 for apoptosis. Bar represents ratios in the densities of bands normalized against β-actin levels in the same sample (n = 5 mice/group). (**E**) Lung homogenates were tested for MPO activity by colorimetric assay which are represented as bar a graph of two independent experiments (n = 5 mice/group).

**Fig 8:**
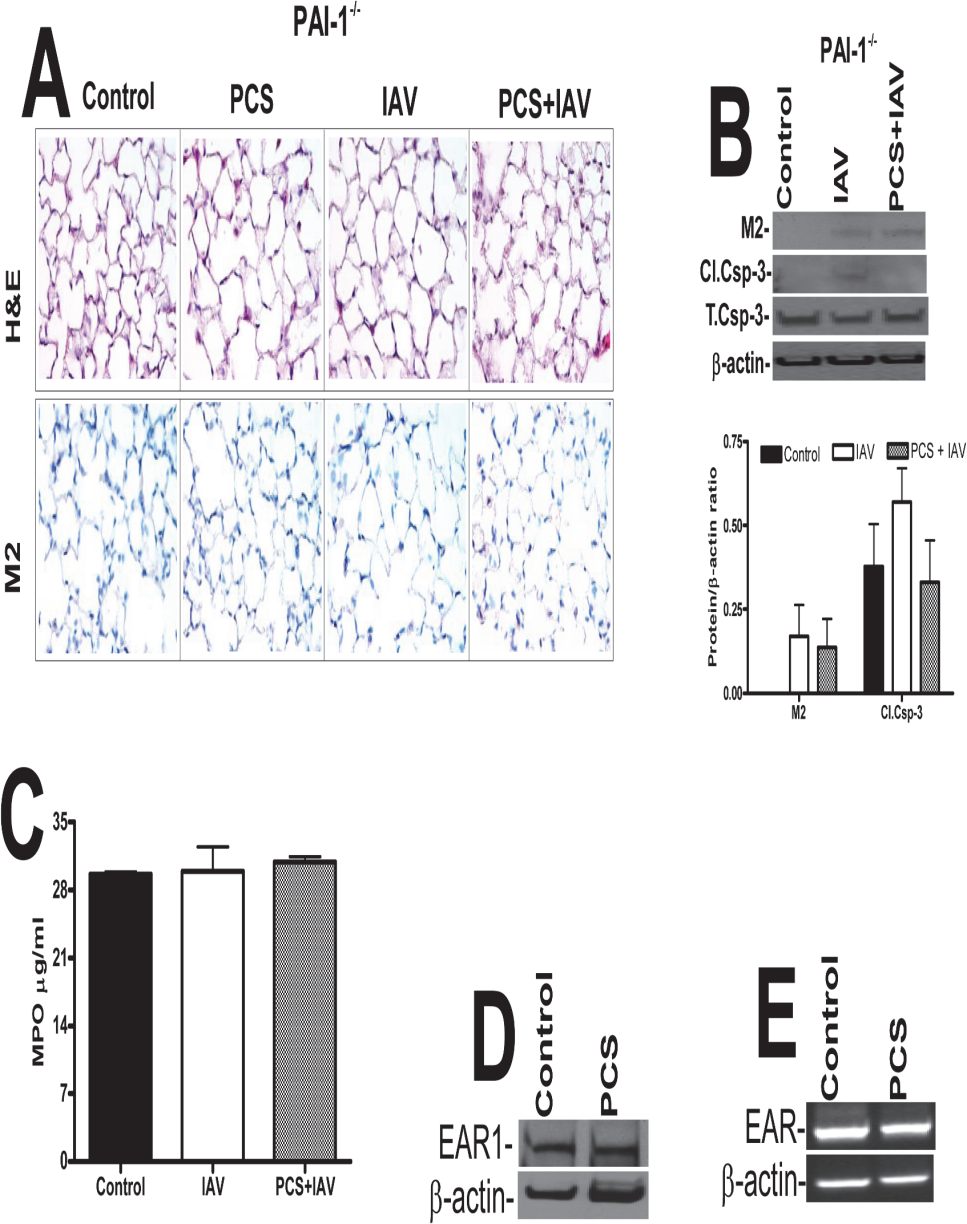
Mice lacking PAI-1 expression resist type II AEC apoptosis. Mice in ambient AIR or exposed to PCS were treated with 50 μl saline or purified IAV in saline via intranasal instillation. One week after IAV infection, the mice were sacrificed. (**A**) Lung sections were subjected to H & E and IHC staining for M2 antigen. Representative fields from 1 of 3 sections per subject are shown at X 400 magnification (n = 5 mice/group). (**B**) Lung homogenates were immunoblotted for changes in IAV M2 and active caspase-3antigens to assess severity of IAV infection and apoptosis respectively. The plot represents ratios in the densities of bands normalized against β-actin levels in the same sample (n = 5 mice/group). (**C**) Lung homogenates were tested for MPO activity by colorimetric assay and represented as a bar graph of two independent experiments (n = 5 mice/group). (**D**) Lung homogenates from the mice in ambient AIR or exposed to PCS for 20 weeks were immunoblotted for changes in EAR1 with β-actin antibody as a loading control. (**E**) Total RNA from the mice exposed to AIR or PCS was analyzed for EAR and β-actin mRNA (n = 5 mice/group).

Further quantification was done using a hemagglutination assay, which revealed a significant increase in the viral titer of lung homogenates of WT mice exposed to PCS and IAV compared to the titer of control mice exposed to ambient air infected with IAV (Fig 7A). However, lung homogenates of PAI-1-deficient mice exposed to PCS plus IAV or ambient air plus IAV demonstrated no significant difference in viral titers. Further, the severity of IAV infection was significantly reduced in PAI-1-deficient mice exposed to PCS compared to WT mice with PCS-induced lung injury. These findings suggest that lung epithelial injury due to PCS-induced PAI-1 predisposes WT mice to more severe IAV infection. Further we analyzed the lung sections of mice exposed to ambient air or PCS with or without IAV for lung inflammation, M2 antigens. PCS exposed mice showed increased parenchymal lung neutrophils and macrophages when compared to control mice in ambient air. We further found that IAV infection increased the level of neutrophils and macrophages, however the accumulation of neutrophils and macrophages in PCS+IAV infected mice was significantly more than those in ambient air exposed to IAV. Consistent with increased susceptibility to IAV infection linked to CS exposure in human subjects [34,35] and in mice [36], we further found that IAV infection induced lung inflammation in mice exposed to PCS is associated with significant increases in M2 antigen levels (Fig 7B). We further analyzed lung sections for p53, PAI-1 and active caspase-3 and all were increased in mice exposed to PCS or IAV, with a maximal response in mice exposed to both PCS and IAV (Fig 7C). As shown in Fig 7D, we found that IAV infection increased p53 and PAI-1 expression in WT mice. These changes were associated with a significant increase in active caspase-3. Analysis of MPO activity further confirmed that there is increased MPO activity in PCS mice infected with IAV compared to control mice in ambient air exposed to IAV or control mice without IAV infection (Fig 7E). Next, in a parallel experiment, to determine whether PAI-1 is involved in AEC apoptosis and severity of IAV infection, we exposed both WT and PAI-1-deficient mice to PCS and infected with IAV as described above after which the responses were compared with mice breathing ambient air which were then exposed to IAV. H & E staining of lung section of PAI-1-deficient mice showed minimal damage to the lung architecture. M2 antigen levels in the lung sections of PAI-1-deficient mice were low indicating resistance to IAV infection (Fig 8A). As shown in Fig 8B, we also found significantly less active caspase-3 in the lung homogenates of PAI-1-deficient mice indicating less IAV-induced lung inflammation compared to WT mice (Fig 7C). PCSE and/or IAV infection also failed to increase in MPO activity in PAI-1-deficient mice (Fig 8C) compared to the corresponding responses in WT mice (Fig 7E). PCSE also failed to suppress EAR1 protein and mRNA expression in PAI-1-deficient mice (Fig 8D and 8E), which is otherwise reduced in WT mice after PCSE injury (Fig 4B).

## Discussion

Chronic CSE results in a series of inflammatory and organizational responses that contribute to airway remodeling and emphysematous changes that are often associated with COPD [37,38]. The damaging effect of CSE/PCSE is a major risk factor throughout the world [39]. AEC apoptosis occurs early in the pathophysiologic events triggered by CSE and is a prelude to the cascade of events terminating in severe disease [40,41]. COPD affects millions of people world-wide with acute exacerbations occurring primarily as a result of viral and bacterial infections.

The inflammatory responses within the lungs occur as a consequence of chronic exposure of AEC to CS through accumulation of inflammatory cells including neutrophils and macrophages. Neutrophils are the major inflammatory cells recruited to the injured lungs in response to IL-8, which are primarily produced by AECs. Neutrophils produce elastase, matrix metalloproteinase’s (MMPs) and cathepsins, all of which often cause increased AEC apoptosis and tissue damage during lung inflammation including that associated with PCSE/CSE. The literature further suggests that neutrophils attract macrophages to the sites of injury [42–44]. Increased accumulation of macrophages is observed in the lungs of mice with PCSE, which promotes lung remodeling via elaboration of MMP-9 and other proteases.

Analysis of the lung sections of patients with COPD revealed that apoptotic AECs in COPD lungs also express increased levels of p53 and PAI-1 and reduced SP-C. The literature documents a critical role of increased p53 in the regulation of AEC apoptosis [16–18, 21]. We previously found that p53 induces expression of PAI-1 in AECs, which in turn contributes to apoptosis [16, 17, 21]. We also found that mice lacking either p53 or PAI-1 resist PCSE-induced lung injury. In addition, inhibition of p53-mediated induction of PAI-1 by inhibiting p53 expression using CSP peptide or competitively blocking p53 interaction with specific PAI-1 mRNA sequences inhibits PAI-1 expression and apoptosis of AECs [16, 17, 21].

PAI-1 has been strongly implicated in the fibrinolytic defect that characteristically occurs in animals and humans with a variety of lung injuries [45, 46] and is induced in the plasma of smokers [15]. The findings we now report extend understanding of the role of PAI-1 in lung inflammation induced by PCSE and indicate that PAI-1 regulates the extent of epithelial and overall lung inflammation associated with PCSE. Here we report that both p53 and PAI-1 expression are induced in AECs by PCSE, that PAI-1 is a downstream mediator of p53-induced lung inflammation and that PAI-1 augments lung inflammation associated with PCSE. The literature [47] suggests that inhibition of apoptosis of neutrophils as well as suppression of phagocytosis of apoptotic neutrophils by increased PAI-1 contributes to their accumulation in injured lungs leading to inflammation. Consistent with these observations, we found that PAI-1-deficient mice resist inflammation that is otherwise increased in WT mice after PCSE-induced lung injury. While we did not find evidence of emphysematous change in our model due to the short period of PCSE, we found that PCSE-induced inflammatory changes included increased lung macrophages and neutrophils and an increased LL protein content.

IAV is a major respiratory pathogen that can cause severe viral pneumonia and which can be complicated by secondary bacterial pneumonia. It has been demonstrated that chronic CS exposure increases the incidence and severity of IAV infection, as indicated by increased viral titers in mice with CSE [34, 35, 36, 48, 49]. AECs are the primary targets for both CS and IAV infection as they are exposed to outer environment. Our data show that IAV infection induces both p53 and PAI-1 expression and apoptosis in AECs. This is consistent with and extends an earlier report where IAV induced apoptotic cell death was causally linked to IAV-induced p53 expression [50, 51]. Based on a number of reports, apoptosis of AECs may promote IAV replication [52,53]. The present study shows that PCS exposure and IAV infection additively increase lung inflammation due to induction of PAI-1 expression. It is unclear how increased PAI-1 during PCSE-induced lung inflammation augments severity of IAV infection. Our studies also demonstrate that increased PAI-1 due to PCSE inhibits EAR1, a member of the RNase A which has potent bactericidal, helminthotoxic, and antiviral properties [54, 55]. Increased IAV infection found after PCSE appears to be associated with suppression of host defense protein, EAR1 by increased PAI-1 in AECs. These studies to our knowledge are the first, to demonstrate that PAI-1 induced during PCSE-induced lung inflammation potentiates IAV-induced cell death.

In summary, this report documents that increased p53 and PAI-1 expression and apoptosis in AECs occur with accumulation of leukocytes in the lungs of patients with COPD, a disease that is promoted by CSE/PCSE. p53 and PAI-1 expression and apoptosis were likewise increased in AECs as was lung inflammation in WT mice. Those lacking p53 or PAI-1 resisted AEC apoptosis and lung inflammation, demonstrating that upregulation of PAI-1 by p53 is central to the pathogenesis of epithelial injury in PCSE. Epithelial apoptosis was reversible by interventions that block increased p53 and PAI-1 in AECs, including CSP or by competitive inhibition of the binding of p53 to PAI-1 mRNA with p53-binding 3’UTR sequences of PAI-1 mRNA. IAV infection increases p53, PAI-1 and apoptosis in AECs with pulmonary inflammation and additively worsened lung inflammation induced by PCSE. Viral burden was directly associated with increased PAI-1 levels in transgenic PAI-1 overexpressing mice and reduced in those lacking PAI-1. Our findings document that PAI-1 expression is increased by PCSE, promotes lung inflammation, AEC apoptosis and exacerbates lung inflammation induced by subsequent exposure to IAV.
